# Tumoricidal Efficacy of Artesunate-Eluting Microsphere-Based Multimodal Therapy in Patient-Derived Colorectal Tumoroids

**DOI:** 10.34133/bmr.0311

**Published:** 2026-01-27

**Authors:** Sarah Helmueller, Farzaneh Vafaeinik, Xinxin Song, Shanghee Lee, Dong-Hyun Kim, Alexandra Gangi, Yong J. Lee

**Affiliations:** ^1^ Department of Biomedical Sciences, Cedars-Sinai Medical Center, Los Angeles, CA 90048, USA.; ^2^ Department of Surgery, UT Southwestern Medical Center, Dallas, TX 75390, USA.; ^3^ Department of Radiology, Feinberg School of Medicine, Northwestern University, Chicago, IL, USA.; ^4^ Robert H. Lurie Comprehensive Cancer Center, Northwestern University, Chicago, IL, USA.; ^5^ Department of Biomedical Engineering, University of Illinois at Chicago, Chicago, IL, USA.; ^6^ Department of Biomedical Engineering, McCormick School of Engineering, Northwestern University, Evanston, IL, USA.; ^7^ Department of Surgery, Cedars-Sinai Medical Center, Los Angeles, CA 90048, USA.

## Abstract

Colorectal cancer (CRC) is a lethal disease characterized by its propensity to metastasize to distant organs. Despite advances in surgery and chemotherapy, CRC remains a major clinical challenge, with high recurrence rates following treatment. The complexity of CRC is further compounded by the limitations of current preclinical models, which often fail to accurately recapitulate the human tumor microenvironment. This underscores the need for improved experimental systems to evaluate novel therapeutic strategies. This study investigates a multimodal second-line treatment strategy using a 3-dimensional (3D), patient-derived CRC tumoroid model that more faithfully mimics the in vivo tumor microenvironment. We evaluated the therapeutic efficacy of a combinatorial approach integrating recombinant human tumor necrosis factor-related apoptosis-inducing ligand (rhTRAIL), artesunate-eluting microspheres (ART-EMs), and mild hyperthermia at 42 °C using a water bath. rhTRAIL selectively induces apoptosis in CRC tumoroids, ART-EMs impose ferroptotic stress, and hyperthermia enhances the crosstalk between these mechanisms. This multitargeted approach is designed to trigger synergistic cell death through the convergence of apoptotic and ferroptotic signaling pathways. Synergistic interactions among rhTRAIL, ART-EMs, and hyperthermia were demonstrated using propidium iodide staining assay, immunoblotting assay, TUNEL (terminal deoxynucleotidyl transferase dUTP nick end labeling) assay, JC-1 assay, and dichlorofluorescein assay. Our findings indicate that the multimodal treatment induces greater tumor cell death than individual monotherapies, primarily through amplification of death signaling pathways in tumoroids. The integration of rhTRAIL, ART-EMs, and hyperthermia represents a promising second-line therapeutic strategy for CRC. By harnessing apoptosis–ferroptosis synergy within a clinically relevant 3D model, this approach has the potential to reduce recurrence and improve patient outcomes.

## Introduction

Colorectal cancer (CRC) originates in the large intestine, specifically the colon or rectum, and it metastasizes frequently to distant organs, with about half of patients developing metastatic disease after at least 5 years post-diagnosis [1]. CRC is the third most commonly diagnosed cancer and the second leading cause of cancer-related death worldwide [1–3]. While chemotherapy, surgery, and targeted immunotherapies have improved short-term outcomes for patients, particularly when CRC is detected early [1–4], recurrence after treatment remains common. Moreover, up to 50% of CRC patients develop drug resistance, underscoring the urgent need for more effective therapeutic strategies [1–3].

One promising avenue for second-line treatment involves targeting the apoptotic cell death pathway through cell membrane death receptors [5–10]. Recombinant human tumor necrosis factor-related apoptosis-inducing ligand (rhTRAIL) selectively triggers programmed cell death in cancer cells by binding to death receptors 4 and 5, while sparing normal tissues [5–11]. rhTRAIL is particularly attractive in CRC second-line treatments because of the microscopic characteristics of tumors after surgical resection [12,13]. Theoretically, rhTRAIL could be used to selectively target microscopic cancer cells by injecting the drug into the tumor area after surgical resection of CRC with the goal of reducing recurrence. However, rhTRAIL’s antitumor efficacy and short half-life have limited its clinical efficacy [6,8,9,14]. These limitations are important to consider, as well as taking into account CRC’s heterogenous tumor tissue composition, which makes these tumors more difficult to treat with single monotherapies [15,16]. To combat the difficulties in using rhTRAIL as a monotherapy, our studies have outlined rhTRAIL as a second-line treatment strategy in combination with other drugs [5–7,10]. There are successful studies that demonstrate rhTRAIL’s tumoricidal effect can be enhanced when combined with drugs that induce ferroptosis, an iron-dependent, non-apoptotic form of cell death driven by lipid peroxidation [5–7,10]. Notably, ferroptosis-induced endoplasmic reticulum (ER) stress synergizes with apoptotic pathways to promote robust cancer cell killing [5–10]. Artesunate (ART), a derivative of artemisinin, is a potent inducer of both ferroptosis and apoptosis, making it a strong candidate for combination therapies [17–21]. Moreover, nanomedicine offers substantial potential for improving therapeutic outcomes [22,23]. In this study, to optimize delivery and localize ART’s therapeutic effect, we developed ART-eluting microspheres (ART-EMs), which accumulate preferentially in capillaries when injected around the tumor site, slowly releasing ART to tumor cells over time [23]. In parallel, mild hyperthermia has been shown to enhance rhTRAIL-induced apoptosis in tumor models [24]. Our prior work demonstrated that hyperthermia amplifies ART–rhTRAIL synergy in HCT116 colon carcinoma and BxPC-3 pancreatic cancer cells [19]. Specifically, heating cells in a water bath at 42 °C for 1 h increased oxidative and ER stress, thereby sensitizing cells to treatment [25]. Building on these findings, this multimodal strategy should be tested in models that more faithfully replicate CRC patient tumors, considering physiological gradients, morphology, and microenvironment, to robustly assess therapeutic efficacy.

Historically, 2-dimensional (2D) cancer cell models have served as foundational tools in preclinical studies evaluating the efficacy of anticancer treatments [11]. While these models offer simplicity and reproducibility, they fall short in encapsulating the spatial and biological complexity of tumors in the human body. A key limitation of monolayer 2D cultures is their inability to accurately mimic the in vivo tumor microenvironment, in both cellular architecture and biological behavior [26]. Conventional 2D models typically rely on a single cell type, resulting in altered morphology, polarity, and gene expression, while neglecting stromal, immune, and endothelial components that critically shape tumor progression and therapeutic response [26]. Furthermore, monolayer cultures are uniformly exposed to drugs, which can overestimate treatment efficacy compared with in vivo conditions. To overcome these 2D cell culture limitations, there has been a shift toward patient-derived 3-dimensional (3D) tumor models, which more faithfully reproduce the architecture and microenvironment of in vivo tumors and provide greater predictive value for therapeutic outcomes [11]. To investigate more effective therapies to treat CRC, we have employed patient-derived 3D CRC tumoroids. In vivo, these tumoroids exist within a complex 3D network of extracellular matrix (ECM), neighboring cells, and vasculature [26]. Such models more accurately capture the features of the tumor microenvironment, including gradients of nutrients, oxygen, and drug penetration, thereby offering a physiologically relevant platform to evaluate therapeutic efficacy [26,27]

In this study, we employed a 3D patient-derived colorectal adenocarcinoma tumoroid model to evaluate a multimodal therapy combining rhTRAIL, ART-EMs, and mild hyperthermia. Our findings revealed synergistic crosstalk between apoptotic and ferroptotic pathways, leading to enhanced tumor cell death and highlighting this multimodal approach as a promising second-line therapeutic strategy for CRC patients.

## Materials and Methods

### Chemicals and reagents

To produce recombinant human TRAIL (rhTRAIL), a human TRAIL cDNA fragment (amino acids 114 to 281) obtained by reverse transcription polymerase chain reaction was cloned into a pET-23d plasmid (Novagen, Madison, WI), and His-tagged TRAIL protein was purified using the Qiagen Express Protein Purification System (Qiagen, Valencia, CA). For the fabrication of ART-EMs, all reagents and solvents were commercially obtained and used without further purification. Poly(d,l-lactide-co-glycolide) (PLGA; lactide:glycolide ratio, 50:50), polyvinyl alcohol (PVA; 89 to 98 kDa, >99% hydrolyzed), dichloromethane, dimethyl sulfoxide (DMSO), and methanol were purchased from Sigma-Aldrich (WI, USA), and ART was obtained from TCI America (OR, USA).

### Manufacturing of ART-EM

ART-EMs were prepared using an emulsion and solvent evaporation method, the same as that reported in our previous work [23]. ART (10 mg) was dissolved in 200 μl of DMSO and combined with 100 mg of PLGA and 3 mg of iron oxide nanoparticles (IONPs) dissolved in 4 ml of dichloromethane [28]. IONPs were synthesized via thermal decomposition. The mixture was vortexed for 1 min to form the primary emulsion, which was then injected into an aqueous solution containing 2 wt% PVA. Emulsification was performed using a homogenizer at 1,500 RPM for 5 min. The organic solvent was evaporated at room temperature for 2 h. ART-EMs were collected by centrifugation at 2,000 RPM for 5 min, washed 3 times with deionized water, lyophilized, and stored at –20 °C. Morphology and size distribution were assessed using an ECHO Revolve phase microscope (ECHO, CA, USA) and scanning electron microscopy (SEM; Hitachi S-4800, Tokyo, Japan). Loaded ART was dissolved in >99% methanol and analyzed by liquid chromatography–tandem mass spectrometry (LC–MS/MS) using a SCIEX QTRAP 6500+ system (SCIEX, MA, USA) with UV detection at 210 nm employed for peak identification. A standard calibration curve was prepared using known concentrations of ART (2.5 to 10,000 ng/ml), and the amount of ART loaded in the ART-EMs was determined based on this calibration. The loaded IONP content was determined by inductively coupled plasma mass spectrometry (ICP-MS; Thermo iCAP Q). Loading contents were calculated as: (mass of ART or IONP / total mass of ART-EM) × 100; loading efficiency were calculated as: (mass of ART or IONP / feeding mass of ART or IONP) × 100.

### Isolation of tumor tissue

This study was approved by the Institutional Review Board of Cedars-Sinai Medical Center (STUDY00002365), and all participants provided written informed consent. Fresh colon adenocarcinoma tissue samples were obtained from surgeries performed by Dr. Alexandra Gangi at Cedars-Sinai Medical Center. Tumor tissues were stored in Servator B Storage Solution (Global Transplant Solutions, University of Wisconsin, USA) in 50-ml tubes at 4 °C for up to 24 h prior to processing.

To isolate tumor cells, tissue samples were transferred to a large petri dish containing ice-cold Dulbecco’s phosphate buffered saline without Ca^2+^ and Mg^2+^ (DPBS), washed to remove blood and debris, and minced into small pieces using sterile scissors and forceps. The minced tissue was transferred to a Falcon cell strainer over a 50-ml tube and further dissociated by grinding through the strainer using a 5-ml syringe plunger with cold DPBS to facilitate transfer. The collected tissue was centrifuged at 1,000 × *g* for 5 min, and the supernatant was aspirated.

The pellet was resuspended in 5 ml of ACK Blood Lysis Buffer and incubated at room temperature for 10 to 15 min to remove red blood cells. This lysis step was repeated 1 to 2 times until tissue appeared white. Samples were then washed 3 to 4 times with freshly prepared 2 mM EDTA chelation buffer (2% sorbitol, 1% sucrose, 1% BSA fraction V, and 1× Gentamicin/Amphotericin in DPBS, sterile filtered). After centrifugation at 1,000 × *g* for 5 min, tissues were incubated on a horizontal orbital shaker for 1 h on ice, followed by additional washes 3 to 4 times with ice-cold chelation buffer without EDTA.

The resulting tissue pellets were resuspended in 15 to 20 ml of digestion buffer (Dulbecco’s Modified Eagle’s Medium [DMEM] supplemented with 2.5% fetal bovine serum [FBS], 1 unit/ml penicillin, 1 μg/ml streptomycin, 2.5 ng/ml amphotericin B, 200 U/ml type IV collagenase, and 125 μg/ml type II dispase) and incubated in a T25 flask for 2 h at 37 °C. After digestion, the suspension was filtered through a small Falcon cell strainer into a 50-ml tube, centrifuged, and washed with ice-cold chelation buffer. The isolated tumor cells were then used for subsequent culture.

### Establishment of tumoroid formation

Matrigel (1 to 2 ml) was thawed on ice. Complete Cell Culture Medium was prepared, consisting of DMEM/F-12 + GlutaMAX supplemented with R-spondin 1 (500 ng/ml), Noggin (100 ng/ml), EGF (50 ng/ml), A83-01 (0.2 μM), Y-27632 dihydrochloride (10 μM), B27 (50×), and 1.25 mM N-acetylcysteine. The complete medium was mixed with the thawed Matrigel at a ratio of approximately 2:1 (Matrigel:medium).

Isolated tumor cells were resuspended in the Matrigel mixture and passed through a Flowmi Cell Strainer into a clean 50-ml tube. Next, 90 μl of the cell–Matrigel suspension was pipetted into the center of each well of a prewarmed 12-well plate. The plate with the cell–Matrigel domes were incubated for 15 min at 37 °C, then carefully inverted and incubated for an additional 5 min. Finally, 1 ml of complete cell culture medium was gently added to each well to fully submerge the Matrigel and tissue, and the plate was incubated at 37 °C.

The tumoroids formed under our culture conditions typically ranged from 120 to 200 μm, with occasional smaller tumoroids around 90 μm and larger tumoroids reaching approximately 270 μm. These sizes were achieved after about 5 to 7 days of tumoroid growth after seeding from the single cell level. These values are estimated from our microscopy images. Consistency in size was promoted by seeding a defined number of cells per well, which encouraged uniform aggregation and reproducible tumoroid formation across experiments.

### Passaging tumoroid embedded culture

For passaging, the tumor-containing Matrigel dome was first collected from the 12-well plate by pipetting up and down with a P1000 tip to break it up. The Matrigel fragments, along with the medium, were transferred into a 15-ml tube and centrifuged at 1,000 × *g* for 5 min at 2 to 8 °C. The supernatant was removed, and the tumoroid pellet was resuspended in 1 ml of trypsin containing Y-27632 dihydrochloride (10 μM) and incubated at 37 °C for 5 min. After trypsin digestion, approximately 2 ml of DMEM was added to the cells, and the suspension was centrifuged again at 1,000 × *g* for 5 min. The resulting pellet was resuspended in diluted Matrigel (ratio ~1:2, complete medium to Matrigel), and 100 μl of the suspension was pipetted into the center of each well of a prewarmed 12-well plate. The plate was incubated for 15 min at 37 °C, then inverted and incubated for an additional 5 min. Finally, 1 ml of complete cell culture medium was gently added to each well, and the plate was returned to 37 °C for incubation.

### Storage and recovery of tumoroids

For cryopreservation of tumoroids, the Matrigel dome was first collected from the 12-well plate by pipetting up and down with a P1000 tip to break it up. The Matrigel fragments and medium were transferred into a 15-ml tube and centrifuged at 1,000 × *g* for 5 min at 2 to 8 °C. The supernatant was discarded, and the tumoroid pellet was resuspended in 1 ml of cold Freezing Medium consisting of DMEM/F-12 + GlutaMAX, supplemented with 10% FBS, 10% DMSO, and Y-27632 dihydrochloride (10 μM). The suspension was transferred into labeled cryovials and stored at –80 °C overnight, followed by long-term storage in liquid nitrogen. For recovery, frozen tumoroids were rapidly thawed in a 37 °C water bath, transferred into 5 ml of DMEM in a 15-ml tube, and centrifuged at 1,000 × *g* for 5 min at 2 to 8 °C. The tumoroid pellet was then resuspended in cold Matrigel and plated under the culture conditions described above.

### PI staining

Tumoroids were cultured in a 12-well plate and treated as indicated. After treatment, tumoroids were washed with PBS to remove medium and debris. A working solution of propidium iodide (PI; Sigma-Aldrich, Cat. No. P4864) was prepared by diluting the stock to a final concentration of 50 μg/ml in PBS and added to each well. Samples were incubated at room temperature in the dark for 30 min and then washed with PBS. Hoechst dye was subsequently added by diluting the stock 1:2,000 in PBS, followed by incubation at room temperature in the dark for 10 to 15 min. After staining, tumoroid morphology and viability were assessed using a fluorescence microscope (ECHO Revolve, ECHO, San Diego, CA, USA).

### TUNEL assay

For apoptosis detection using the terminal deoxynucleotidyl transferase dUTP nick end labeling (TUNEL) method, tumoroids were plated in a 12-well plate and treated as indicated. Following treatment, tumoroids were gently washed once with phosphate-buffered saline (PBS) without disrupting the Matrigel. TUNEL reaction mixture was added, and samples were incubated at 37 °C for 1 h in the dark using the In Situ Cell Death Detection Kit, Fluorescein (Roche, Cat. No. 11684795910), according to the manufacturer’s instructions. After incubation, tumoroids were washed with PBS, and Hoechst dye was added at a 1:2,000 dilution in PBS, followed by incubation at room temperature in the dark for 10 to 15 min. The samples were then washed with PBS and imaged using an ECHO fluorescence microscope.

### siRNA transfection in 3D tumoroids

In order to knock down Chop and Bax protein expression, we transfected tumoroids with siRNA. Chop siRNA (Santa Cruz Biotechnology, Cat. No. sc-35437) and BAX siRNA (Fisher Scientific Cat. No. 4390824) were used at 50 nM concentration for treatment. Prior to adding to samples, siRNA was diluted into Lipofectamine RNAiMAX Transfection Reagent (Fisher Scientific, Cat. No. 13778030) and then further diluted into transfection medium; we used DMEM for best results. Samples were treated with diluted siRNA (50 nM concentration) for 24 h at 37 °C.

### JC-1 assay

To assess mitochondrial membrane potential (ΔΨm), tumoroids were cultured in a 12-well plate and treated as indicated. JC-1 dye (Thermo Fisher Scientific, Cat. No. T3168) was used to evaluate ΔΨm according to the manufacturer’s instructions. The JC-1 assay indicates changes in ΔΨm by using a fluorescent probe, JC-1, which aggregates and fluoresces red in cells with high membrane potential, indicating healthy mitochondria and cells. However, in cells with low membrane potential, or in unhealthy conditions, the JC-1 probe aggregates and fluoresces green. Therefore, the color change from red to green indicated by the JC-1 probe will reflect decrease in ΔΨm, equating a loss of ΔΨm and overall cell health. Following treatment, tumoroids were washed once with PBS and stained with JC-1 dye. After incubation, the tumoroids were washed with PBS, and Hoechst dye was added at a 1:2,000 dilution in PBS, followed by incubation at room temperature in the dark for 10 to 15 min. After a final PBS wash, fluorescence images were captured using an ECHO microscope.

### DCF assay

Reactive oxygen species (ROS) production was analyzed using the DCF assay, which relies on the cell-permeant reagent DCF-DA (dichlorodihydrofluorescein diacetate). Inside the cell, DCF-DA is converted to the nonfluorescent compound DCFH, which is subsequently oxidized by ROS into the fluorescent compound, DCF. Tumoroids were cultured in a 12-well plate and treated as indicated. Following treatment, DCF-DA dye (Cat #D6883, Sigma-Aldrich) was added according to the manufacturer’s instructions. After incubation, tumoroids were washed with PBS, and Hoechst dye was added at a 1:2,000 dilution in PBS, followed by incubation at room temperature in the dark for 10 to 15 min. After a final PBS wash, fluorescence images were captured using an ECHO microscope.

### Mean fluorescence intensity analysis

For cytotoxicity analysis using immunofluorescence data, mean fluorescence intensity (MFI) of images captured during Annexin V, JC-1, TUNEL, and Hoechst staining was quantified using ImageJ software. Images were first split into RGB channels via “Image”<“Color”<”Split Channels” and analyzed in grayscale. MFI values were measured from 3 randomly selected fields for each experimental condition. For JC-1 analysis, JC-1 Red, JC-1 Green, and Hoechst MFIs were quantified using ImageJ. The JC-1/Hoechst ratio was calculated by dividing the JC-1 Red or Green MFI by the corresponding Hoechst MFI in each field. For Annexin V assays, the MFI of Annexin V (Green channel, early apoptosis), PI (Red channel, late apoptosis), and Hoechst staining was quantified. Ratios of Annexin V/Hoechst and PI/Hoechst were calculated and averaged over triplicate samples. Similarly, for TUNEL assays, MFI of TUNEL and Hoechst stains was measured, and the TUNEL/Hoechst ratio was calculated by dividing the TUNEL MFI by the Hoechst MFI. The resulting values represent the average over 3 independent samples.

### Western blot analysis and antibodies

Immunoblotting was performed as previously described [13]. The following antibodies were used: anti-PARP-1 (#9532), anti-caspase-3 (#9668), anti-caspase-8 (#9746), and anti-caspase-9 (#7237) (Cell Signaling Technology, Beverly, MA); and anti-actin, goat anti-rabbit IgG-HRP, and goat anti-mouse IgG-HRP (Santa Cruz Biotechnology, Santa Cruz, CA).

### Combination index analysis

Combination index (CI) analysis was performed using CompuSyn software (ComboSyn, Inc., Paramus, NJ, USA), a widely used tool developed by Dr. Dorothy Chu in 2005 for evaluating drug interactions across multiple treatments, doses, and combinations. CompuSyn applies the median-effect principle of the mass-action law and the CI equation to quantify drug interactions. The median-effect equation defines the dose–effect relationship using 2 parameters: the median-effect dose (*D*_m_), representing potency, and the slope (*m*), reflecting the dose–response curve shape. The combination index equation (CIE) extends this model to classify interactions as synergistic (CI < 1), additive (CI = 1), or antagonistic (CI > 1). Unlike traditional methods requiring extensive curve fitting, CompuSyn uses a “top-down” approach that requires fewer dose points and leverages internal reference parameters for predictive simulations. In this study, CI values were calculated for each treatment combination of ART-EMs and rhTRAIL, with or without mild hyperthermia. CI values above 1 indicate antagonism, values between 0.9 and 1.10 indicate additive effects, 0.85 to 0.9 suggest slight synergy, 0.3 to 0.7 indicate moderate synergy, and values below 0.3 indicate strong synergy.

### Statistical analysis

Statistical analysis was performed using 1-way and 2-way analysis of variance followed by Sidak’s or Tukey’s multiple comparisons test as indicated using GraphPad Prism 8 software. *P* values less than 0.05 were defined as statistically significant. *P* values are indicated as follows: *P < 0.05; **P < 0.01; ***P < 0.001; ****P < 0.0001.

## Results

### rhTRAIL-induced cytotoxicity in tumoroids

**Fig. 1.**
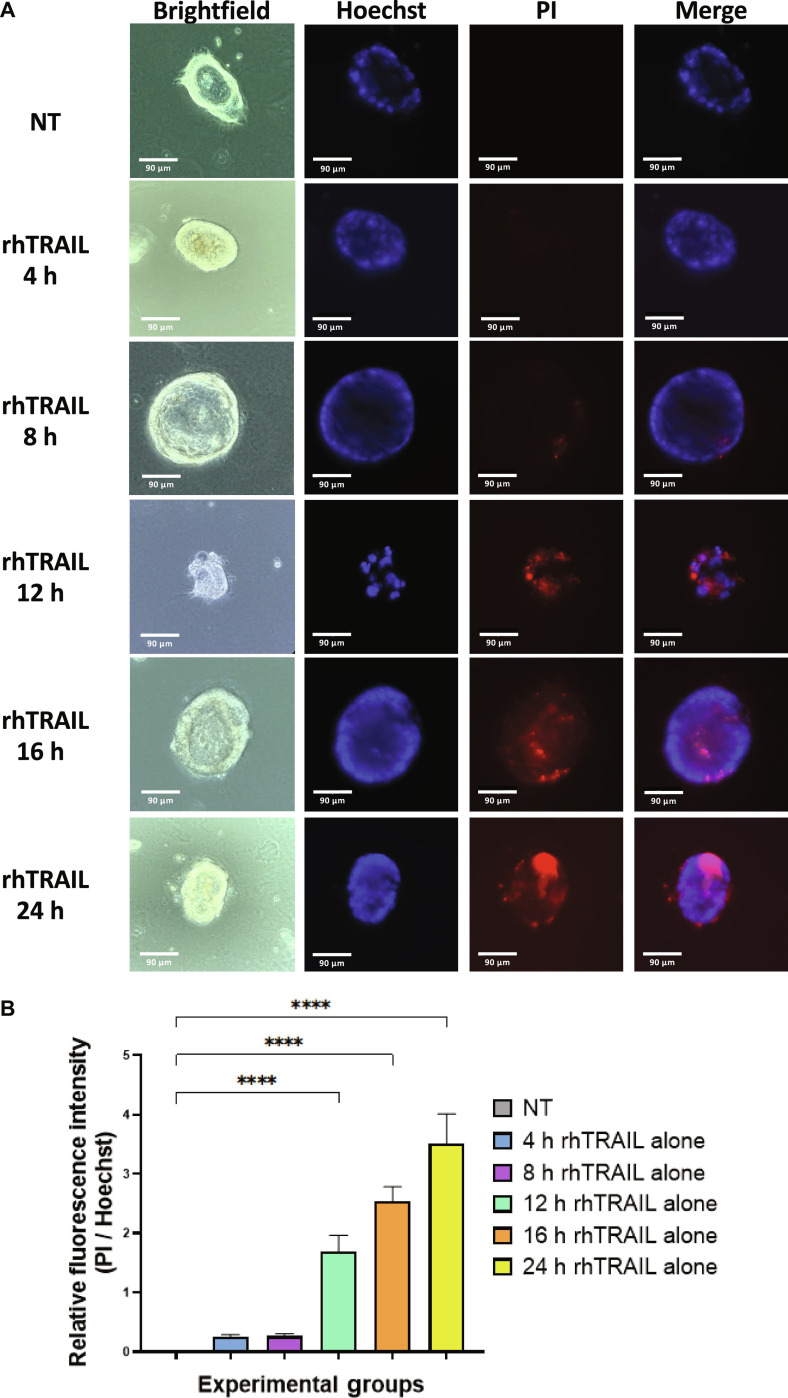
rhTRAIL treatment induces cytotoxicity in patient-derived colorectal cancer tumoroids. Tumoroids were treated with 2 ng/ml rhTRAIL for 4, 8, 12, 16, and 24 h. (A) Following treatment, PI staining was performed to assess plasma membrane damage, and tumoroids were imaged using an ECHO Revolve microscope. Brightfield and fluorescence images were captured, with Hoechst staining used to visualize cell nuclei. NT: untreated control; stained and analyzed after 24 h to validate that the apoptosis observed from rhTRAIL treatment is not due to oxygen or nutrient diffusion limitations. (B) Relative fluorescence intensity was quantified using ImageJ by calculating the mean fluorescence intensity (MFI) ratio of PI to Hoechst staining. Bar plots represent the PI/Hoechst ratio, with error bars showing mean ± SD from triplicate experiments. Statistical significance:****P < 0.0001.

To evaluate the effects of rhTRAIL on 3D patient-derived CRC tumoroids, we first assessed tumoroid viability during a time-course treatment with 2 ng/ml rhTRAIL. Tumoroids embedded in Matrigel in a 12-well plate were treated with 2 ng/ml rhTRAIL for 4, 8, 12, 16, and 24 h, and cytotoxicity was assessed using PI staining to detect for membrane damage in tumoroids. PI signals began to appear after 12 h of treatment, indicating that rhTRAIL-induced cytotoxicity requires at least 12 h to manifest in 3D tumoroid models (Fig. 1A). Therefore, PI-positive staining indicates cells undergoing late apoptosis or necrosis, where the plasma membrane is permeable. Brightfield (BF) imaging reveals clear morphological shifts consistent with rhTRAIL-induced cytotoxicity. Control (NT) tumoroids retained compact, spherical architecture with uniform boundaries, whereas the “rhTRAIL 24 h” sample displayed surface blebbing and uneven morphology, indicative of localized cytotoxic effects and compromised structural integrity (Fig. 1A). By 24 h, cytotoxicity was further increased, as evidenced by the highest mean, or relative fluorescence intensity (MFI) compared to the control (Fig. 1B).

### Hyperthermia enhances rhTRAIL-induced cytotoxicity in tumoroids

**Fig. 2.**
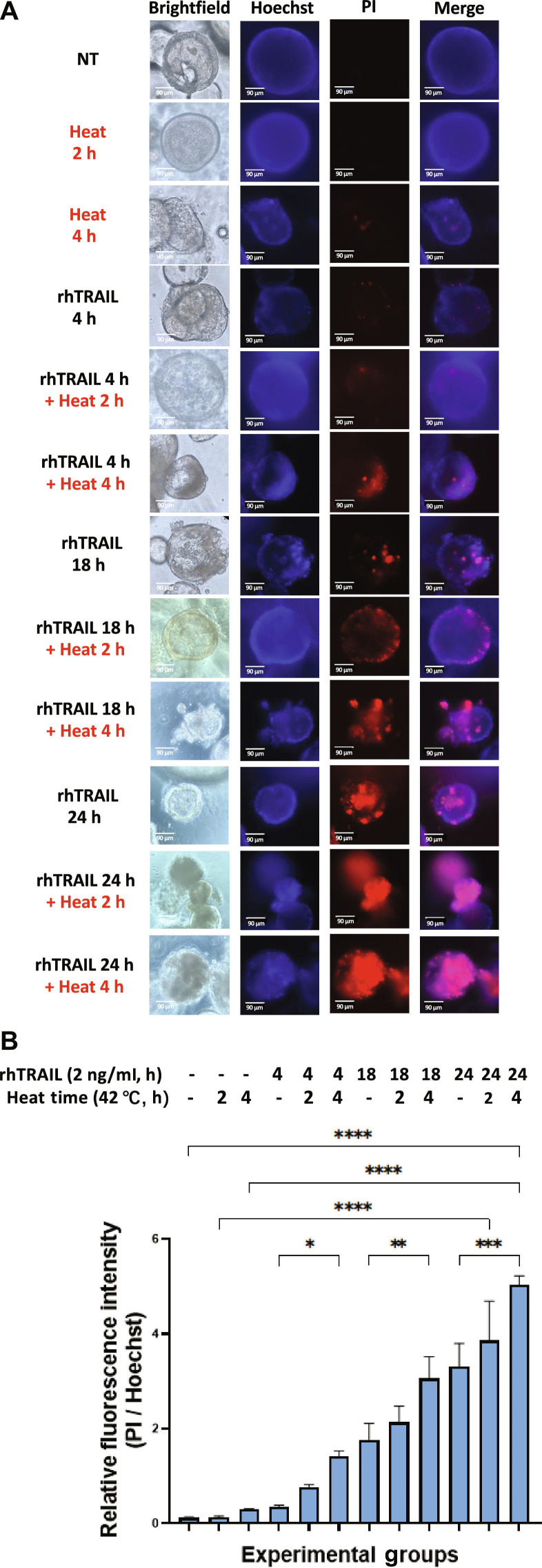
Hyperthermia enhances rhTRAIL treatment-induced cytotoxicity in patient-derived colorectal cancer tumoroids. Tumoroids were treated with 2 ng/ml rhTRAIL for 4, 18, or 24 h. Some samples were additionally subjected to hyperthermia in a 42 °C water bath for 2 or 4 h before returning to a 37 °C incubator to complete rhTRAIL treatment. (A) Following treatment, PI staining was performed to assess plasma membrane damage, and tumoroids were imaged using an ECHO Revolve microscope. Brightfield and fluorescence images were captured, with Hoechst staining used to visualize cell nuclei. NT: untreated control; stained and analyzed after 24 h to validate that the apoptosis observed from rhTRAIL treatment is not due to oxygen or nutrient diffusion limitations. (B) Relative fluorescence intensity was quantified using ImageJ by calculating the MFI ratio of PI to Hoechst staining. Bar plots represent the PI/Hoechst ratio, with error bars showing mean ± SD from triplicate experiments. Statistical significance: *P < 0.05; **P < 0.01; ***P < 0.001; ****P < 0.0001.

After observing that rhTRAIL induces cytotoxicity in 3D tumoroids with at least 12 h of treatment, we next assessed whether hyperthermia could enhance rhTRAIL’s effects. Tumoroids in a 12-well plate were treated with or without 2 ng/ml rhTRAIL for 4, 18, or 24 h, combined with either 2 or 4 h of heat at 42 °C. In 3D tumoroids, treatment with rhTRAIL alone for 4 h did not produce detectable cytotoxicity (Fig. 1). However, exposing tumoroids to 4 h of rhTRAIL treatment, with 2 h (out of the 4 h) under hyperthermic conditions, resulted in increased cytotoxicity, as indicated by PI staining (Fig. 2A). Extending the heating duration to 4 h further amplified cytotoxic effects compared to 2 h of hyperthermia (Fig. 2A). The hyperthermia-enhanced rhTRAIL-induced cytotoxicity was even more pronounced in samples treated for 18 and 24 h (Fig. 2A). Additionally, BF imaging revealed morphological changes consistent with hyperthermia-enhanced rhTRAIL-induced cytotoxicity (Fig. 2A). Control (NT) tumoroids maintained a smooth, spherical shape, whereas tumoroids treated with “rhTRAIL 24 h”, “rhTRAIL 24 h + Heat 2 h”, and “rhTRAIL 24 h + Heat 4 h” exhibited extensive membrane blebbing, break-off of tumoroid fragments, and the formation of darker, aggregated regions, which are characteristic of cellular stress and death (Fig. 2A).

### ART-EMs synergistically enhance rhTRAIL-induced cytotoxicity in tumoroids

**Fig. 3.**
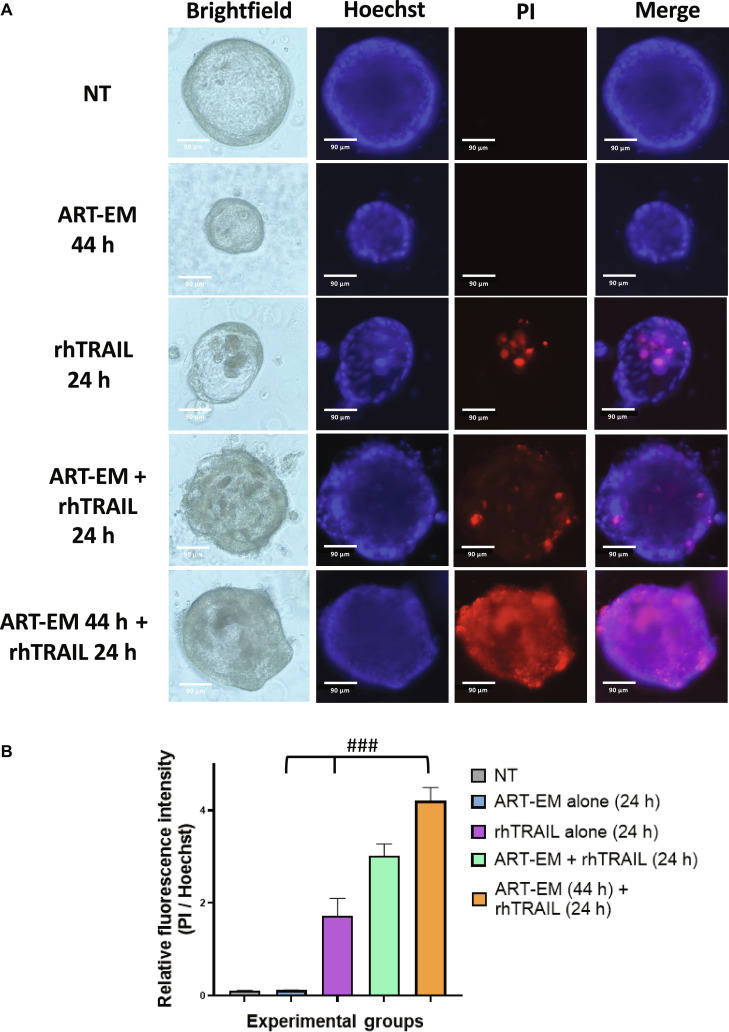
ART-EMs combined with rhTRAIL synergistically promotes cytotoxicity in tumoroids. Tumoroids were treated with 50 μM ART-EM alone for 24 h (ART-EM 24 h), 2 ng/ml rhTRAIL alone for 24 h (rhTRAIL 24 h), a combination of 50 μM ART-EM and 2 ng/ml rhTRAIL for 24 h (ART-EM + rhTRAIL 24 h), or pretreated with 50 μM ART-EM for 20 h followed by 2 ng/ml rhTRAIL for 24 h in the presence of ART-EM (ART-EM 44 h + rhTRAIL 24 h). (A) Following treatment, PI staining was performed to assess plasma membrane damage, and tumoroids were imaged using an ECHO Revolve microscope. Brightfield and fluorescence images were captured, with Hoechst staining used to visualize cell nuclei. NT: untreated control; stained and analyzed after 68 h to validate that the apoptosis observed is not due to oxygen or nutrient diffusion limitations. (B) Relative fluorescence intensity was quantified using ImageJ by calculating the MFI ratio of PI to Hoechst staining. Bar plots represent the PI/Hoechst ratio, with error bars showing mean ± SD from triplicate experiments. Combination index (CI) analysis was performed to assess synergy between multiple drug doses. CI: ###, strong synergy.

Having observed that hyperthermia enhances cytotoxicity of rhTRAIL in CRC tumoroid models, we next investigated whether the ferroptosis-inducing agent ART-EMs could further potentiate rhTRAIL-induced cytotoxicity [17]. Detailed physicochemical properties of ART-EMs have been reported in our previous studies [23], including an average particle size of 16 μm and ART loading content of 8% with a loading efficiency of 57%. To assess the most effective order while treating with ART-EMs and rhTRAIL, samples were either treated with 50 μM ART-EMs at the same time as 2 ng/ml rhTRAIL for 24 h, or the sample was pretreated with 50 μM ART-EMs for 20 h, and then treated with 2 ng/ml rhTRAIL in the presence of ART-EMs for another 24 h. Samples were assayed with PI staining after treatment. Pretreatment with 50 μM ART-EMs for 20 h, followed by combined treatment with ART-EMs and rhTRAIL, resulted in markedly increased cytotoxicity in the tumoroids compared to all other samples (Fig. 3A). BF images also reflect an increase in cytotoxicity in this sample as there is a darkening of the cells and cellular membrane disruption (Fig. 3A). Combinatorial index values calculated between the mean or relative fluorescence values (MFI) also indicate strong synergy during ART-EM and rhTRAIL combination treatment where ART-EMs were pretreated for 24 h (Fig. 3B).

### Combination therapy using ART-EMs, rhTRAIL, and hyperthermia potentiates cytotoxic and apoptotic effects

**Fig. 4.**
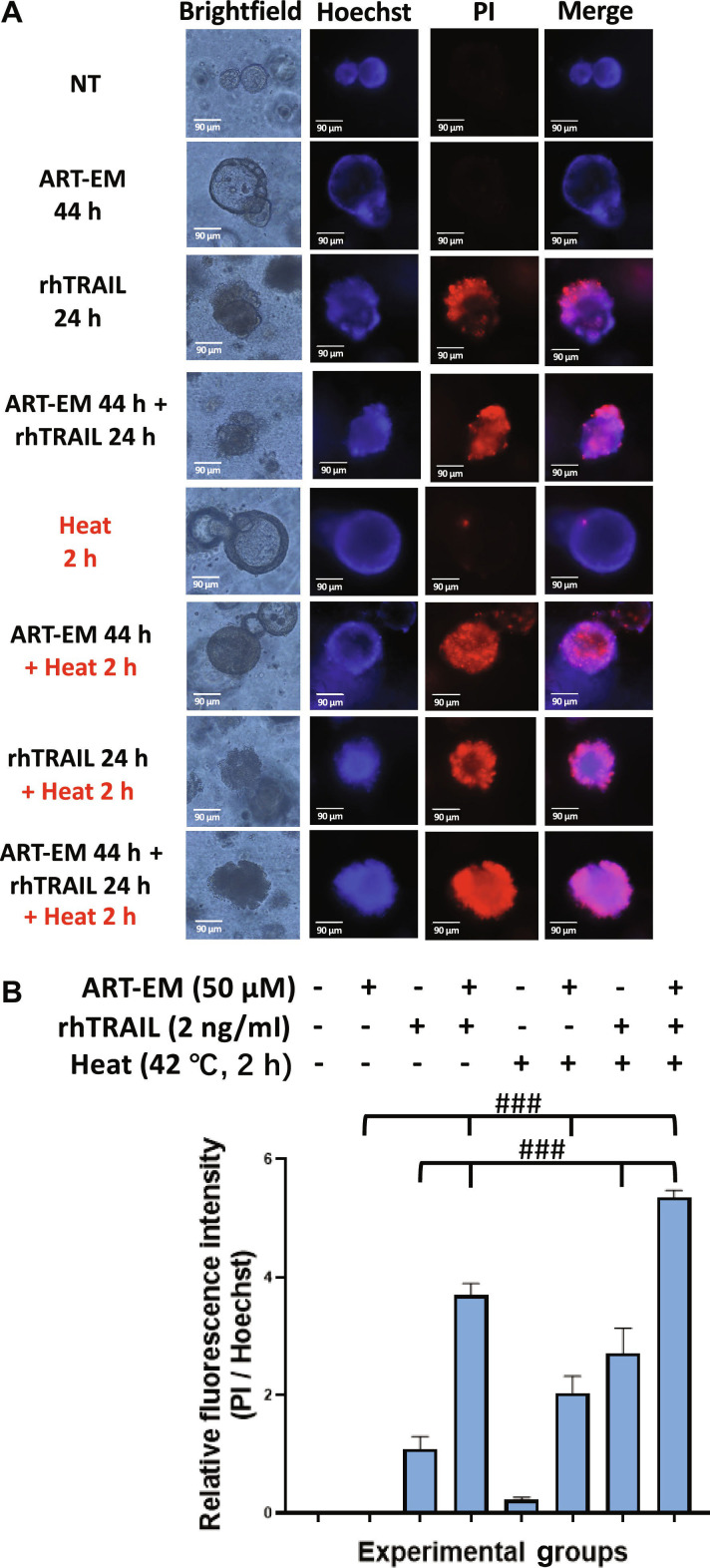
Hyperthermia promotes multimodal (ART-EM + TRAIL) treatment-induced cytotoxicity in tumoroids. Tumoroids were treated with 50 μM ART-EM alone for a total of 44 h (ART-EM 44 h), 2 ng/ml rhTRAIL alone for 24 h (rhTRAIL 24 h), pretreated with 50 μM ART-EM for 20 h followed by 2 ng/ml rhTRAIL for 24 h in the presence of ART-EM (ART-EM 44 h + rhTRAIL 24 h), heated at 42 °C for 2 h without rhTRAIL or ART-EM (Heat 2 h), pretreated with 50 μM ART-EM for 20 h followed by 2 h of 42 °C heat in the presence of ART-EM for another 22 h (ART-EM 44 h + Heat 2 h), heated at 42 °C for 2 h in the presence of rhTRAIL for a total of 24 h (rhTRAIL 24 h + Heat 2 h), or pretreated with 50 μM ART-EM for 20 h followed by treatment with 2 ng/ml rhTRAIL, then heated for 2 h at 42 °C, and then put back in the incubator for a total of another 24 h in the presence of ART-EM (ART-EM 44 h + rhTRAIL 24 h + Heat 2 h). (A) After treatment, PI staining assay was used to assess for DNA damage in the tumoroid by analysis under an ECHO Revolve microscope. Brightfield and fluorescence images were captured, and Hoechst staining was used to stain cell nuclei. NT: untreated control. (B) ImageJ program was used to analyze the relative fluorescence intensity of each of the samples by calculating the MFI of the Hoechst stain compared to the PI stain. The bar plot represents the ratio of PI stain fluorescence intensity to Hoechst stain fluorescence, with error bars representing the mean ± SD from triplicate experiments. CI analysis was performed to assess synergy between multiple drug doses. CI: ###, strong synergy.

Next, we evaluated the cytotoxic effects of all 3 treatments in combination. In the multimodal treated sample, tumoroids were pretreated with 50 μM ART-EMs for 20 h and then exposed to 2 ng/ml rhTRAIL, with a subset subjected to 2 h of hyperthermia at 42 °C, followed by incubation at 37 °C for the remaining 24 h rhTRAIL treatment period in the presence of ART-EMs. PI staining revealed that the combination of ART-EMs, rhTRAIL, and hyperthermia induced the highest levels of cytotoxicity in the tumoroids compared to all other treatment groups (Fig. 4A). Again, referencing BF images reflect an increase in cytotoxicity in the multimodal-treated sample “ART-EM 44 h + rhTRAIL 24 h + Heat 2 h” as there is marked darkening of the tumoroid and blebbing of the tumoroid membrane (Fig. 4A). Additionally, analysis of the relative fluorescence intensity revealed a strong synergistic interaction, as indicated by CI values demonstrating pronounced synergy during the multimodal treatment (Fig. 4B).

**Fig. 5.**
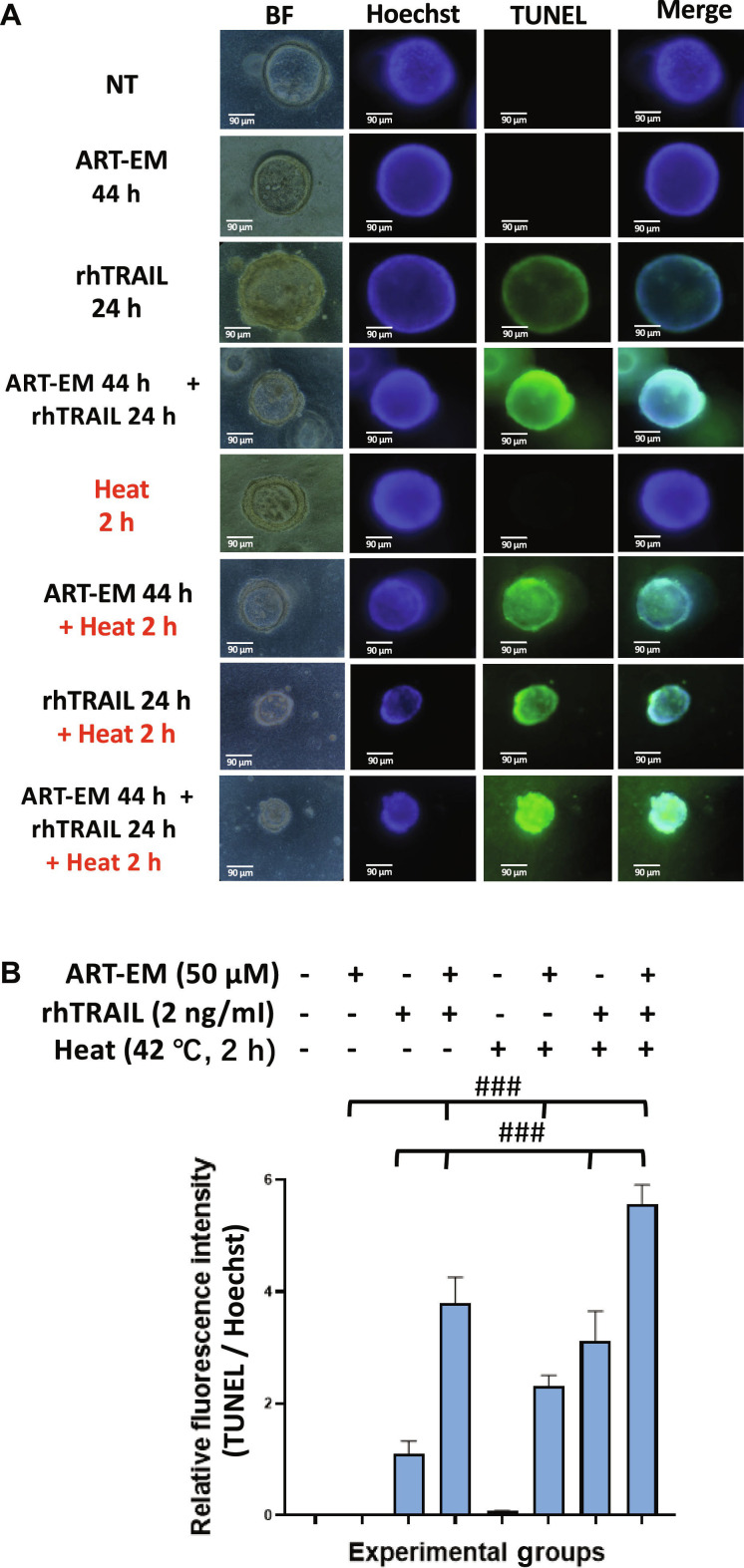
Multimodal treatment induces apoptosis in tumoroids. Tumoroids were treated with 50 μM ART-EM alone for a total of 44 h (ART-EM 44 h), 2 ng/ml rhTRAIL alone for 24 h (rhTRAIL 24 h), pretreated with 50 μM ART-EM for 20 h followed by 2 ng/ml rhTRAIL for 24 h in the presence of ART-EM (ART-EM 44 h + rhTRAIL 24 h), heated at 42 °C for 2 h without rhTRAIL or ART-EM (Heat 2 h), pretreated with 50 μM ART-EM for 20 h followed by 2 h of 42 °C heat in the presence of ART-EM for another 22 h (ART-EM 44 h + Heat 2 h), heated at 42 °C for 2 h in the presence of rhTRAIL for a total of 24 h (rhTRAIL 24 h + Heat 2 h), or pretreated with 50 μM ART-EM for 20 h followed by treatment with 2 ng/ml rhTRAIL, then heated for 2 h at 42 °C, and then put back in the incubator for a total of another 24 h in the presence of ART-EM (ART-EM 44 h + rhTRAIL 24 h + Heat 2 h). (A) Immediately after treatment, TUNEL staining assay was performed on treated tumoroid samples and fluorescent images were examined under an ECHO fluorescence microscope. Brightfield (BF) and fluorescence images were captured, and Hoechst staining was used to stain cell nuclei. NT: untreated control. (B) ImageJ program was used to analyze the relative fluorescence intensity of each of the samples, by calculating the MFI of the Hoechst stain compared to the TUNEL stain. The bar plot represents the ratio of TUNEL stain fluorescence intensity to Hoechst stain fluorescence, with error bars representing the mean ± SD from triplicate experiments. CI analysis was performed to assess synergy between multiple drug doses. CI: ###, strong synergy.

As mentioned, the PI staining assay demonstrated varying levels of induced cytotoxicity across the samples. However, to further, and more specifically, assess the mechanism of cytotoxicity, we performed the TUNEL assay to detect DNA fragmentation and therefore determine if apoptosis occurred during multimodal treatment in 3D tumoroids. Fluorescein isothiocyanate (green) fluorescence was elevated in tumoroids treated with ART-EMs and rhTRAIL, and this signal was further exacerbated by exposing ART-EM + rhTRAIL-treated samples to hyperthermic conditions (Fig. 5). These results consistently indicate that the combination therapy effectively promotes apoptosis.

### Multimodal therapy-induced apoptosis using ART-EMs, rhTRAIL, and hyperthermia is dependent on Chop and Bax

**Fig. 6.**
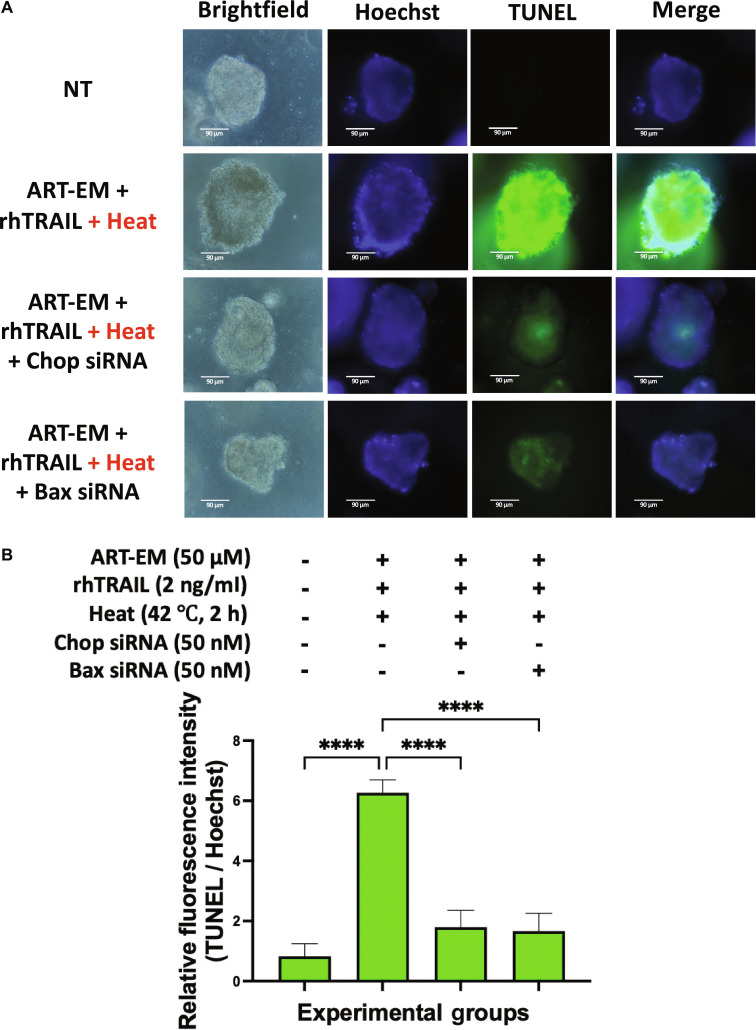
Multimodal treatment-induced apoptosis is dependent on Chop and Bax. Tumoroids were pretreated with 50 nM of Chop siRNA or 50 nM of Bax siRNA following manufacturer’s instructions. After 24 h of siRNA treatment, tumoroids were pretreated with 50 μM ART-EM for 20 h followed by treatment with 2 ng/ml rhTRAIL, then heated for 2 h at 42 °C, and then put back in the incubator for a total of another 24 h in the presence of ART-EM. (A) Immediately after treatment, TUNEL staining assay was performed on treated tumoroid samples and fluorescent images were examined under an ECHO fluorescence microscope. Brightfield (BF) and fluorescence images were captured, and Hoechst staining was used to stain cell nuclei. NT: untreated control. (B) ImageJ program was used to analyze the relative fluorescence intensity of each of the samples, by calculating the MFI of the Hoechst stain compared to the TUNEL stain. The bar plot represents the ratio of TUNEL stain fluorescence intensity to Hoechst stain fluorescence, with error bars representing the mean ± SD from triplicate experiments.

Since we found that apoptosis was induced in tumoroids treated with the multimodal therapy, we next wanted to investigate if the mechanism had dependence on well-characterized pro-apoptotic protein, Bax, which is a key enzyme in the mitochondrial-associated apoptotic-signaling-cascade [5–7,10,23,25]. Along with Bax, we also wanted to investigate if ER stress was an important part of the multimodal therapy-induced apoptotic mechanism by silencing the expression of ER stress-associated and apoptosis-promoting transcription factor, Chop, in tumoroid samples [5–7]. To elucidate the roles of Bax and Chop in the multimodal treatment-induced apoptosis mechanism, we pretreated samples with 50 nM Chop and Bax siRNA, respectively, per the manufacturer’s instructions. After siRNA transfection, tumoroids were treated with the multimodal treatment as described, and then the TUNEL assay was used to assess apoptotic activity. The sample treated with the combination of ART-EMs, rhTRAIL, and heat demonstrate strong green fluorescence, indicating high levels of apoptosis. However, the sample treated with Chop siRNA, as well as the sample treated with Bax siRNA, both have diminished levels of green fluorescence, indicating that silencing Chop and Bax protein expression inhibited apoptosis during multimodal treatment in tumoroids (Fig. 6A). MFI analysis reflects this result (Fig. 6B).

### The multimodal treatment causes polarization in the mitochondrial membrane potential and the generation of ROS

**Fig. 7.**
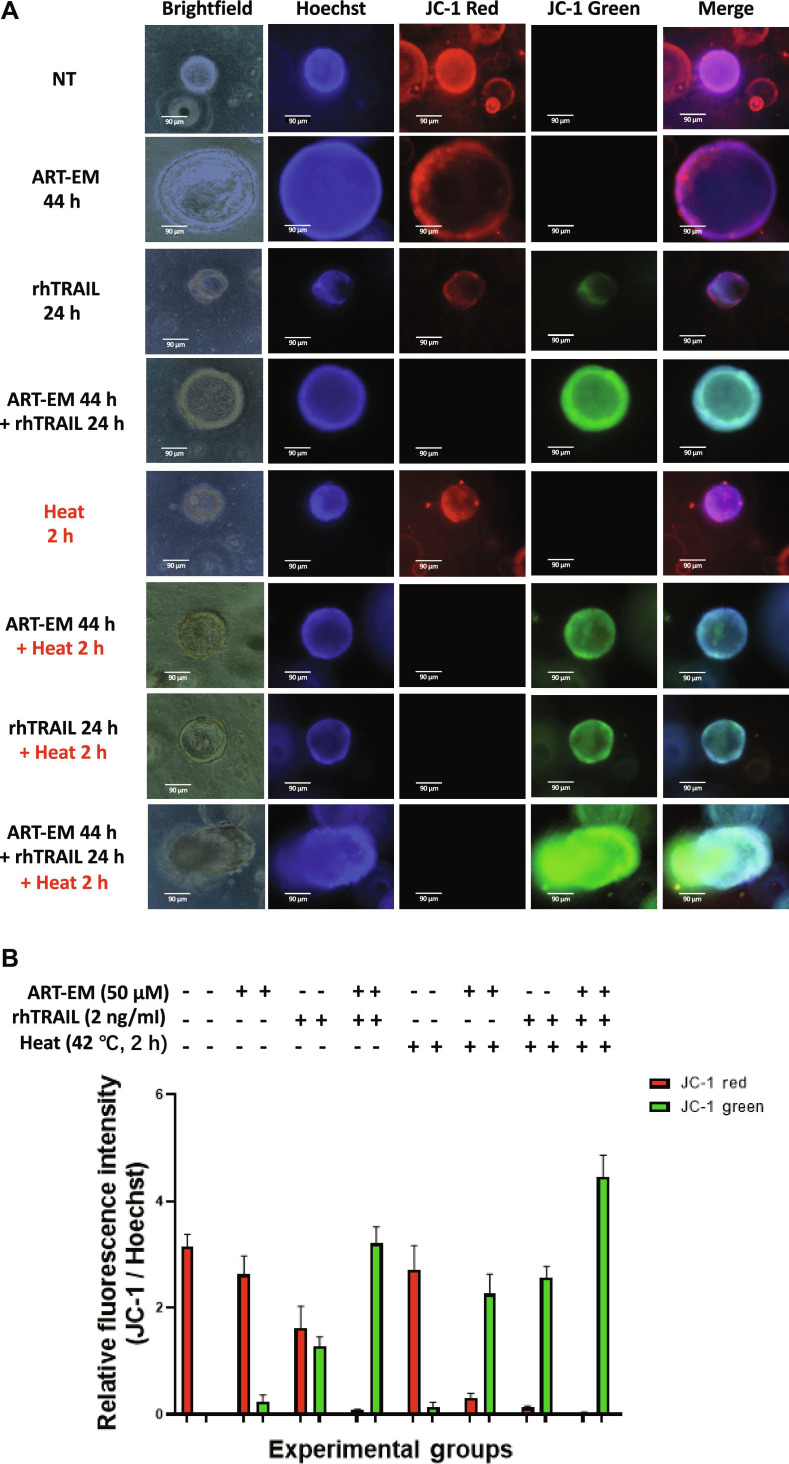
The multimodal treatment causes polarization in the mitochondrial membrane potential. Tumoroids were treated with 50 μM ART-EM alone for 44 h (ART-EM 44 h), 2 ng/ml rhTRAIL alone for 24 h (rhTRAIL 24 h), pretreated with 50 μM ART-EM for 20 h followed by 2 ng/ml rhTRAIL for 24 h in the presence of ART-EM (ART-EM 44 h + rhTRAIL 24 h), heated at 42 °C for 2 h without rhTRAIL or ART-EM (Heat 2 h), pretreated with 50 μM ART-EM for 20 h followed by 2 h of 42 °C heat in the presence of ART-EM for another 22 h (ART-EM 44 h + Heat 2 h), heated at 42 °C for 2 h in the presence of rhTRAIL for a total of 24 h (rhTRAIL 24 h + Heat 2 h), or pretreated with 50 μM ART-EM for 20 h followed by treatment with 2 ng/ml rhTRAIL, then heated for 2 h at 42 °C, and then put back in the incubator for a total of another 24 h in the presence of ART-EM (ART-EM 44 h + rhTRAIL 24 h + Heat 2 h). (A) JC-1 staining assay was performed immediately after treatment concluded and results were analyzed using an ECHO fluorescence microscope. Brightfield and fluorescence images were captured, and Hoechst staining was used to stain cell nuclei. NT: untreated control. (B) ImageJ program was used to analyze the relative fluorescence intensity of each of the samples, by calculating the MFI of the Hoechst stain compared to the JC-1 red or green stain. The bar plot represents the ratio of JC-1 stain fluorescence intensity to Hoechst stain fluorescence, with error bars representing the mean ± SD from triplicate experiments.

Mitochondria are known to be key targets of apoptotic agents, and we examined if mitochondrial function was affected during combination treatment in 3D colorectal tumoroid models. The JC-1 assay was performed to assess changes in mitochondrial membrane potential (ΔΨm) (Fig. 7). The JC-1 assay indicates changes in ΔΨm by using a fluorescent probe, JC-1, which aggregates and fluoresces red in cells with high membrane potential, indicating healthy mitochondria, but in cells with low mitochondrial membrane potential and unhealthy conditions, the JC-1 probe aggregates and fluoresces green. Therefore, the color change from red to green indicated by the JC-1 probe demonstrates a decrease in ΔΨm and cell health. Samples treated with the combination of ART-EMs, rhTRAIL, and 2 h of 42 °C hyperthermia exhibited decreased red fluorescence and increased green fluorescence, indicating mitochondrial depolarization and stress associated with cytotoxic activity (Fig. 7).

**Fig. 8.**
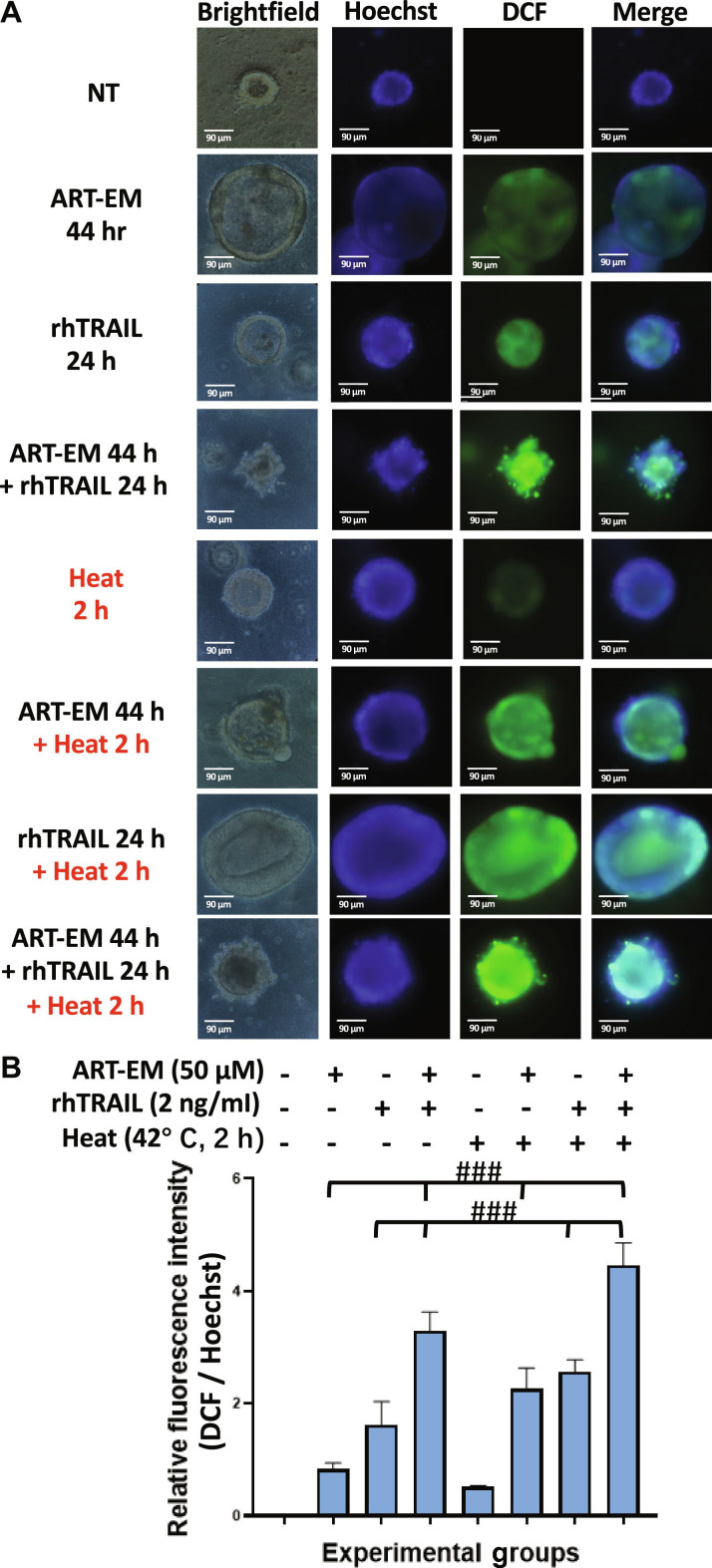
Multimodal treatment leads to the generation of reactive oxygen species in tumoroids. Tumoroids were treated with 50 μM ART-EM alone for 44 h (ART-EM 44 h), 2 ng/ml rhTRAIL alone for 24 h (rhTRAIL 24 h), pretreated with 50 μM ART-EM for 20 h followed by 2 ng/ml rhTRAIL for 24 h in the presence of ART-EM (ART-EM 44 h + rhTRAIL 24 h), heated at 42 °C for 2 h without rhTRAIL or ART-EM (Heat 2 h), pretreated with 50 μM ART-EM for 20 h followed by 2 h of 42 °C heat in the presence of ART-EM for another 22 h (ART-EM 44 h + Heat 2 h), heated at 42 °C for 2 h in the presence of rhTRAIL for a total of 24 h (rhTRAIL 24 h + Heat 2 h), or pretreated with 50 μM ART-EM for 20 h followed by treatment with 2 ng/ml rhTRAIL, then heated for 2 h at 42 °C, and then put back in the incubator for a total of another 24 h in the presence of ART-EM (ART-EM 44 h + rhTRAIL 24 h + Heat 2 h). (A) DCF-DA staining assay was performed immediately after treatment concluded and results were analyzed using an ECHO fluorescence microscope. Brightfield and fluorescence images were captured, and Hoechst staining was used to stain cell nuclei. NT: untreated control. (B) ImageJ program was used to analyze the relative fluorescence intensity of each of the samples, by calculating the MFI of the Hoechst stain compared to the DCF-DA stain. The bar plot represents the ratio of JC-1 stain fluorescence intensity to Hoechst stain fluorescence, with error bars representing the mean ± SD from triplicate experiments. CI analysis was performed to assess synergy between multiple drug doses. CI: ###, strong synergy.

Another hallmark of cellular stress and mitochondrial dysfunction is the generation of ROS. To determine whether the combinatorial treatment increased ROS levels, we performed the DCF-DA assay. Results show that tumoroids treated with ART-EMs, rhTRAIL, and hyperthermia produced the highest amounts of ROS compared to all other groups (Fig. 8).

**Fig. 9.**
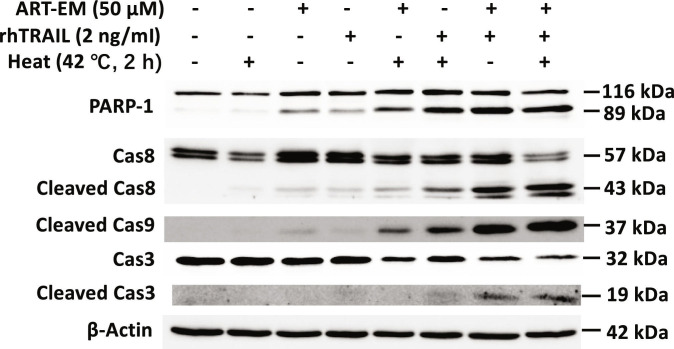
ART-EMs combined with hyperthermia promotes TRAIL-induced apoptosis in tumoroids. Tumoroids were either heated at 42 °C for 2 h without rhTRAIL or ART-EM (Heat 2 h), treated with 50 μM ART-EM alone for 44 h (ART-EM 44 h), treated with 2 ng/ml rhTRAIL alone for 24 h (rhTRAIL 24 h), pretreated with 50 μM ART-EM for 20 h followed by 2 h of 42 °C heat in the presence of ART-EM for another 22 h (ART-EM 44 h + Heat 2 h), heated at 42 °C for 2 h in the presence of rhTRAIL for a total of 24 h (rhTRAIL 24 h + Heat 2 h), pretreated with 50 μM ART-EM for 20 h followed by 2 ng/ml rhTRAIL for 24 h in the presence of ART-EM (ART-EM 44 h + rhTRAIL 24 h), or pretreated with 50 μM ART-EM for 20 h followed by treatment with 2 ng/ml rhTRAIL, then heated for 2 h at 42 °C, and then put back in the incubator for a total of another 24 h in the presence of ART-EM (ART-EM 44 h + rhTRAIL 24 h + Heat 2 h). After treatment, whole-cell extracts were collected and analyzed with an immunoblotting assay using the indicated antibodies.

Finally, apoptosis was further evaluated by immunoblotting for poly(ADP-ribose) polymerase 1 (PARP-1) and caspase cleavage, canonical markers of apoptotic cell death (Fig. 9). ART-EMs alone induced minimal apoptosis, whereas combining ART-EMs with hyperthermia markedly enhanced caspase activation and PARP-1 cleavage. Similarly, rhTRAIL-induced apoptosis was potentiated by hyperthermia. The greatest apoptotic effect, reflected in high PARP-1 and caspase cleavage, was observed with the combination of ART-EMs, rhTRAIL, and hyperthermia in patient-derived colorectal tumoroids (Fig. 9).

### Schematic diagram of multimodal therapy

**Fig. 10.**
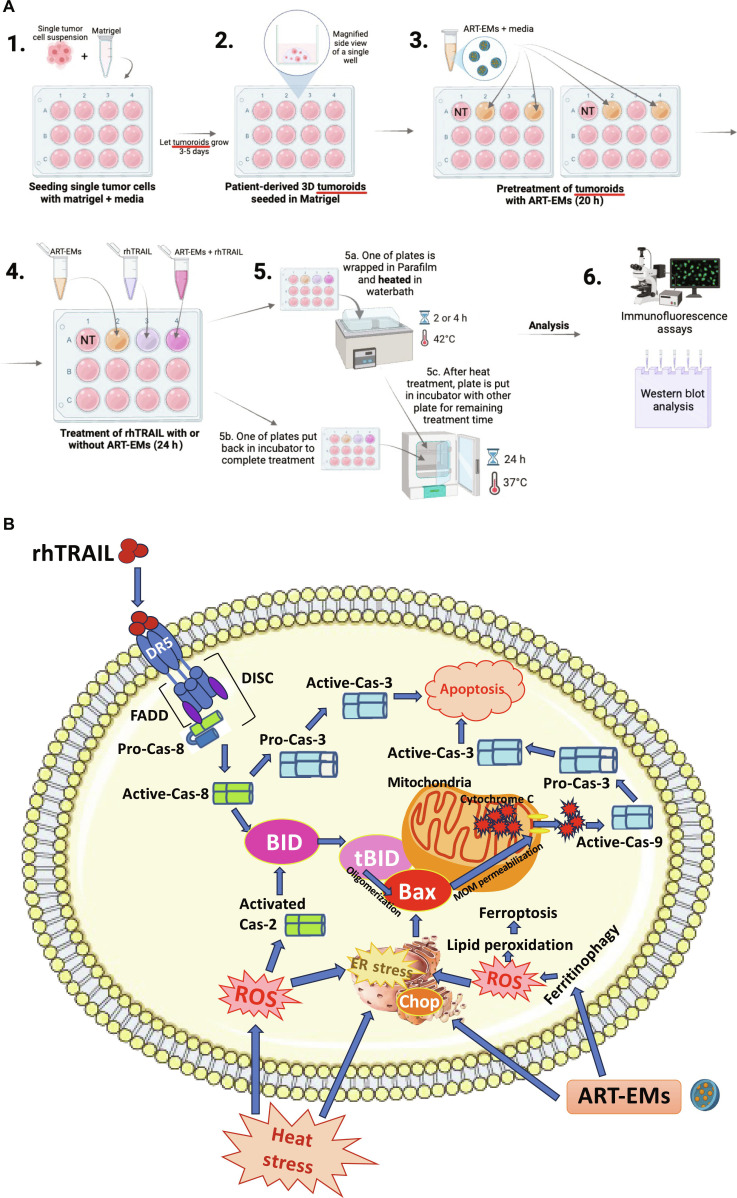
(A) Schematic visually demonstrating the multimodal treatment protocol. (B) Diagram illustrating the synergism between apoptosis and ferroptosis during the hyperthermia-enhanced multimodal therapy utilizing ART-EMs and rhTRAIL.

Figure 10 presents an overview summarizing the key experimental findings. ART-EM inhibits glutathione *S*-transferase, thereby reducing intracellular glutathione levels and activating ER stress responses [5], which subsequently promote mitochondrial-dependent apoptosis through a BID–Bax signaling axis. ART-EM also enhances ferritinophagy-mediated iron release, facilitating Fenton chemistry, ROS accumulation, and iron-dependent lipid peroxidation. Cytokine rhTRAIL selectively binds to DR4 and DR5 on tumoroids, triggering the caspase activation cascade and the truncation of BID. Truncated BID (tBID) then oligomerizes Bax, leading to mitochondrial outer membrane permeabilization, cytochrome c release, activation of caspase-9, and subsequent caspase-3 activation. Activated caspase-3 cleaves downstream targets, including PARP-1, culminating in apoptosis. Mild hyperthermia induces cellular heat stress, resulting in ROS generation and the up-regulation of protective heat shock proteins HSP70 and HSP90. Under these conditions, HSP70 and HSP90 act to buffer the Unfolded Protein Response (UPR). However, when combined with ART-EM-enhanced, rhTRAIL-mediated apoptosis, heat stress can exceed HSP buffering capacity, disrupt proteostasis, and further activate ER stress and UPR signaling. Additionally, hyperthermia-generated ROS may induce low levels of caspase-2 activation, which is markedly amplified under the multimodal hyperthermia-based treatment. Overall, the schematic highlights how hyperthermia augments ART-EM and rhTRAIL-induced apoptosis through ROS amplification and coordinated crosstalk between ER-stress-mediated mitochondrial signaling and the BID–Bax mitochondrial apoptotic pathway.

## Discussion

In this study, we demonstrate that a multimodal treatment strategy combining rhTRAIL, ART-EMs, and mild hyperthermia markedly enhances cytotoxicity and apoptosis in patient-derived CRC tumoroids. Using a physiologically relevant 3D culture system, our results support the hypothesis that targeting apoptotic pathways under stress-enhancing conditions, such as hyperthermia, can produce synergistic tumor cell death. This multimodal approach offers a promising strategy to overcome key clinical challenges in CRC, including treatment resistance and disease recurrence.

Our results demonstrate that rhTRAIL alone exhibited modest cytotoxicity, with cell death detectable only after 12 to 24 h of exposure to rhTRAIL (Fig. 1). In some samples, PI staining appeared concentrated at the tumoroid tip, which likely reflects diffusion-driven gradients in rhTRAIL exposure and the greater permeability of rhTRAIL to the outer tumoroid regions (Fig 1A). Such variation between tumoroids is expected due to structural heterogeneity and microenvironmental differences. Continually, although rhTRAIL alone does induce mild cytotoxicity, the addition of short exposure to hyperthermia significantly amplified rhTRAIL-induced cytotoxicity across multiple time points (Fig. 2). Next, we observed that ART-EMs alone induced limited cytotoxicity but markedly enhanced rhTRAIL-induced cell death when used in combination (Fig. 3), adding to the growing evidence of crosstalk between ferroptotic and apoptotic pathways, particularly via ER and mitochondrial stress signaling [5–7,10,19]. When hyperthermia was included in the ART-EM and rhTRAIL combination treatment, as seen in the protocol outlined in Fig. 10A, tumoroids exhibited the highest levels of cytotoxicity, mitochondrial depolarization, ROS production, and DNA fragmentation, as shown by JC-1, DCF-DA, and TUNEL assays, respectively (Figs. 4, 5, and 7). These findings indicate that hyperthermia not only enhances apoptotic and ER–mitochondrial stress responses but also serves as a catalyst for the mechanistic convergence of these pathways.

Western blot analysis further supports these mechanistic insights. Cleavage of caspase-3, caspase-8, caspase-9, and PARP-1 confirmed activation of both intrinsic and extrinsic apoptotic pathways in response to the triple therapy (Fig. 9). ART-EMs or hyperthermia alone induced minimal cleavage, whereas the addition of rhTRAIL markedly enhanced caspase activation and PARP-1 processing. Furthermore, TUNEL assay analysis of siRNA transfection using Chop and Bax indicates that ER stress and mitochondrial-mediated apoptosis pathways are a central part of the multimodal-induced apoptosis mechanism (Fig. 6). These results indicate that the multimodal treatment synergistically promotes apoptosis by concurrently engaging mitochondrial, death receptor, and ER stress-related cell death pathways (Fig. 10B).

A critical aspect of this study is understanding the mechanistic role of mild hyperthermia in enhancing the synergy between ART-EMs and rhTRAIL. Our recent findings [23,25] identified the pro-apoptotic proteins Bid and Bax as a key molecular mediator facilitating crosstalk between apoptotic and ferroptotic pathways during hyperthermia-enhanced multimodal treatment utilizing ART and rhTRAIL [5,7,23,29]. Specifically, Bid links the extrinsic death receptor pathway to the intrinsic mitochondrial apoptotic pathway, where tBid cleaves and oligomerizes Bax, leading to release cytochrome C from the mitochondria into the cytosol and activation of caspase-9 [23]. We confirmed the importance of Bax in the multimodal treatment in our siRNA experiment, where Bax inhibition in tumoroids reduced the efficacy of the multimodal treatment (Fig. 6). In previous studies, mild hyperthermia alone increased cleavage of caspase-2, which can process Bid into its active form, tBid [25,30]. However, we hypothesized that although hyperthermia alone induces stress signals sufficient to activate caspase-2, the resulting tBid levels are insufficient to trigger apoptosis independently. This is supported by our 3D tumoroid data, where hyperthermia alone caused minimal PARP-1 cleavage, whereas the triple treatment produced robust PARP-1 cleavage (Fig. 9). Collectively, these findings suggest that Bid and Bax serve as consistent mediators in the synergistic interaction among hyperthermia, ART-EMs, and rhTRAIL in both 3D tumoroid models and 2D cell cultures [23,25].

As suggested, hyperthermia may increase cellular susceptibility to both rhTRAIL and ART-EMs by amplifying ER stress and ROS accumulation (Figs. 7 and 8) [25]. When combined with rhTRAIL and ART-EMs, these heat-amplified stressors act synergistically to enhance apoptotic signaling and cell death [23,25,27,30]. As shown in Figs. 7 and 8, the highest levels of mitochondrial depolarization and ROS production were observed during the full triple treatment. Overall, our findings in 3D tumoroid models are consistent with previous observations in 2D cell culture systems [25] and further support the hypothesis that hyperthermia potentiates synergistic apoptosis during combined ART-EM and rhTRAIL therapy.

To better evaluate our multimodal treatment hypotheses, we employed 3D patient-derived tumoroids instead of the 2D cell culture models used in our initial study [11,25,26]. The 3D tumoroid system is essential for capturing therapeutic effects in a context that more accurately reflects in vivo tumor architecture and microenvironment. While 2D cultures are convenient and allow for rapid experimentation, they fail to replicate key tumor features such as nutrient and oxygen gradients, cellular heterogeneity, and stromal interactions, all of which influence drug response [11,26]. By using 3D tumoroids, we observed treatment dynamics that are likely more predictive of clinical outcomes, particularly regarding drug penetration, resistance, and combination effects [4,11,26]. Although 3D tumoroids offer advantages over 2D cultures, further validation in even more complex models is warranted. Future studies could leverage microfluidics-based tumoroid-on-a-chip platforms, which integrate 3D cultures with controlled microenvironments and co-culture with stromal components such as endothelial cells and fibroblasts, or even immune cells [26,27]. Investigating multimodal treatment responses in these advanced models will be critical for successful clinical translation.

Another important consideration in the application of our novel multimodal treatment is that the clinical translation of localized hyperthermia for CRC remains limited because of challenges in achieving targeted and controlled heat delivery to tumors. While hyperthermia is currently being used to treat superficial or hepatic tumors, precision in CRC tumors is complicated by anatomical constraints, heterogeneous vascularization, and variations in tissue thermal conductivity [31–33]. Emerging delivery strategies, including intratumoral administration and hepatic arterial infusion of thermosensitive nanoparticles, such as the ART-EMs proposed in this study, offer promising strategies to enhance localization and generate hyperthermia under an alternating current magnetic field [34,35]. Nonetheless, ensuring uniform heat distribution and preventing injury to adjacent intestinal and vascular tissues remain critical safety considerations and areas of further investigation.

Beyond delivery challenges, localized hyperthermia can modulate the tumor microenvironment. Elevated temperatures can temporarily modulate ECM architecture, reducing matrix stiffness and altering interstitial fluid flow, which can influence mechanotransductive signaling cascades and cellular behaviors such as adhesion, migration, and immune cell recruitment [36,37]. These thermally induced changes to the tumor microenvironment give reason to further investigate hyperthermia in patient-mimetic models, particularly in combination with chemotherapeutics or second-line treatments, to potentiate synergistic effects. Microfluidics-based 3D tumoroid-on-a-chip platforms, which incorporate ECM, immune cells, and vascular architecture, can help study these biothermal and mechanical dynamics [20,23,36,37].

It is also important to recognize potential safety concerns related to off-target effects, as any cytotoxic treatment could affect neighboring healthy tissues. rhTRAIL is generally considered tumor-selective, as it targets death receptors located on cancer cells, making cancer cells more sensitive to TRAIL-induced apoptosis than normal cells [8,9,38]. Hyperthermia at the mild, controlled level as we used in the study (42 °C) can also preferentially stress tumor tissues due to their abnormal vasculature and metabolic profile [29,35,39,40]. Nonetheless, careful dosing and localized delivery would be essential in vivo to minimize any cytotoxic effects on surrounding healthy tissue. Preclinical studies using 3D tumoroids and animal models will be critical to further evaluate safety and efficacy. Together, these studies will help advance localized hyperthermia combined with rhTRAIL and ART-EMs toward safe and effective clinical translation in CRC.

In conclusion, our study demonstrates that the combination of rhTRAIL, ART-EMs, and mild hyperthermia induces synergistic cell death by converging apoptotic mechanisms in 3D patient-derived CRC tumoroids. This multimodal strategy represents a promising second-line therapeutic approach for CRC and merits further preclinical development toward clinical translation.

## Data Availability

Research materials that were generated in the studies including plasmid DNA constructs will be made freely available to the scientific research community as soon as this manuscript has been documented in a publication. Raw data were generated at the Cedars-Sinai Medical Center. Derived data supporting the findings of this study are available from the corresponding author (Y.J.L.) on request.
